# Development of a High-Content Orthopoxvirus Infectivity and Neutralization Assays

**DOI:** 10.1371/journal.pone.0138836

**Published:** 2015-10-01

**Authors:** Irina Gates, Victoria Olson, Scott Smith, Nishi Patel, Inger Damon, Kevin Karem

**Affiliations:** 1 Atlanta Research and Education Foundation, Decatur, Georgia, United States of America; 2 Poxvirus and Rabies Branch, Division of High-Consequence Pathogens and Pathology (DHCPP), National Center for Emerging and Zoonotic Infectious Diseases (NCEZID), Centers for Disease Control and Prevention, Atlanta, Georgia, Unites States of America; University of Texas HSC at San Antonio, UNITED STATES

## Abstract

Currently, a number of assays measure Orthopoxvirus neutralization with serum from individuals, vaccinated against smallpox. In addition to the traditional plaque reduction neutralization test (PRNT), newer higher throughput assays are based on neutralization of recombinant vaccinia virus, expressing reporter genes such as β-galactosidase or green fluorescent protein. These methods could not be used to evaluate neutralization of variola virus, since genetic manipulations of this virus are prohibited by international agreements. Currently, PRNT is the assay of choice to measure neutralization of variola virus. However, PRNT assays are time consuming, labor intensive, and require considerable volume of serum sample for testing. Here, we describe the development of a high-throughput, cell-based imaging assay that can be used to measure neutralization, and characterize replication kinetics of various Orthopoxviruses, including variola, vaccinia, monkeypox, and cowpox.

## Introduction

Variola virus is the etiological agent of smallpox, arguably the most lethal infectious disease of man in history. International efforts, including a world-wide vaccination campaign, resulted in eradication of smallpox in 1980 [1]. Subsequent cessation of routine vaccination caused the global community to become increasingly vulnerable to Orthopoxvirus infections as a result of waning immunity [1]. In recent years, research and development for therapies and safer vaccines have been driven by concerns over use of variola virus as an agent of bioterrorism, or emergence of other Orthopoxvirus infections as public health threats [2, 3]. Stockpiles of vaccine are currently held in many countries, including the United States, in order to vaccinate the populations in the event of a smallpox reintroduction. However, existing vaccines remain unsafe for people in certain risk categories, including infants, pregnant and breastfeeding women, people with immunodeficiency or those who are likely to experience adverse side effects [4]. Research to develop next generation smallpox vaccines requires some measure of immune efficacy in the absence of an animal model susceptible to variola virus infection. As such, these new vaccines will require efficacy testing to show non-inferiority to first generation smallpox vaccines. Studies for efficacy may include animal models using other Orthopoxvirus infection, immune assays to measure induced immunity, and functional assays such as *in vitro* viral neutralization tests. *In vitro* assays traditionally used to monitor humoral immunity include a traditional plaque reduction neutralization test (PRNT) for poxviruses. Traditional PRNT assays have been a benchmark for poxvirus neutralization for decades. However, they are low-throughput, labor-intensive, require a large amount of serum and significant amounts of time to perform ranging between 48–72 hours. Contemporary high-throughput assays have been developed that increase dynamic range, shorten protocol time, decrease serum volume required for assay, and improve reproducibility [5, 6, 7, 8]. However, these newer assays rely on recombinant vaccinia viruses in order to measure viral infection via fluorescent or luminescent methods. Recombination of variola virus is prohibited internationally under the authority of the World Health Organization, forbidding manipulation of variola virus for use in existing high throughput assays [9]. We therefore sought to develop a high-throughput neutralization assay for use with variola virus without the requirement of genetic manipulation. Exploiting the known immunological cross-reactivity between members of Orthopoxviruses, and the availability of commercial polyclonal antibodies against vaccinia virus, we designed infectivity and neutralization assays that do not require use of recombinant viruses and are compatible with different species of the Orthopoxvirus genus. We initially developed high-throughput, high-content infectivity and neutralization assays using vaccinia virus and applied the method to measure infectivity and neutralization of variola, cowpox, and monkeypox viruses.

## Materials and Methods

### Cells, viral stocks, Vaccinia Immune Globulin (VIG) and staining procedure

Vero E6 cells (ATCC CRL1586) were maintained in growth medium containing DMEM with Glutamax (Life Technologies), supplemented with 10% of heat-inactivated FBS (HyClone), 100 units/mL of Penicillin and 100ug/mL of Streptomycin (Life Technologies) and were sub-cultured twice a week using Accumax (Innovative Cell Technologies) as detachment reagent. Cell infections were carried out in medium that contained 2% FBS, 100 units/mL of Penicillin and 100ug/mL of Streptomycin. The following Orthopoxviruses were used in assay development: vaccinia Wyeth, vaccinia Western Reserve, variola Solaimen, monkeypox USA-2003-44, and cowpox Brighton. All work with variola virus was additionally reviewed and approved by the World Health Organization (WHO) Technical Advisory Committee on variola virus research. Viral stocks were generated at different times according to standard laboratory protocols in which Orthopoxviruses were grown in BSC-40 cells, harvested and purified by sedimentation through sucrose cushion. Viral potencies were determined by plaque assay on BSC-40 cells monolayer. VIG (lot # 1730206) was received from Cangene Corporation (Winnipeg, Canada), and used for neutralization assay development experiments.

For imaging assays to detect virus presence, infected cells were fixed with 4% formaldehyde (Pierce, Rockford, IL. USA), followed by permeabilization with 0.5% Triton X-100 (Sigma, St. Louis, MO, USA). Viral presence was detected with rabbit anti-vaccinia polyclonal antibody (Virostat, Portlamd, ME, USA), and counterstained with goat anti-rabbit antibody conjugated to Alexa Fluor 488. DAPI stain was used for identification of cellular nuclei, and HCS Cell Mask Deep Red Cell Stain for staining of cellular cytoplasm (Life Technologies). Images were acquired on the ArrayScan high content instrument (Thermo Scientific), using 5X objective, excitation wavelengths at 350, 488 and 633 nm, and emission data collection at 470, 519, and 665nm, correspondingly. With 5X magnification, the three fluorescent signals were collected from 9 fields per well, which is the maximum number of fields possible to collect at selected magnification. Image analysis was done using Columbus Scope software (Perkin Elmer).

### High Content Infectivity Assay (HC-IA)

**Fig 1:**
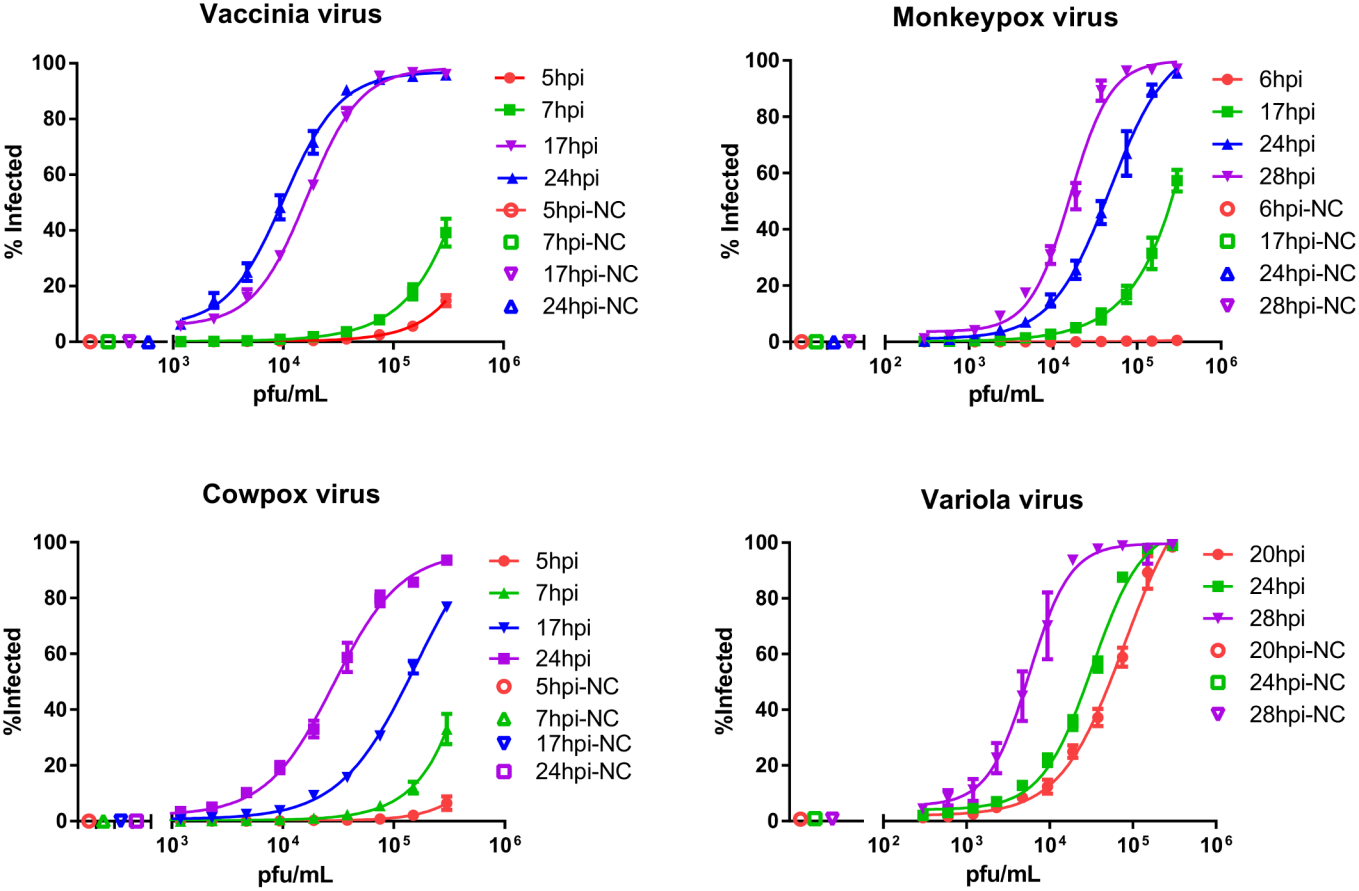
Infection dynamics. Infection dynamics were tested in Vero E6 cells for four different types of Orthopoxviruses. Cells were infected with viral titrations starting from MOI 1 (1.7x10^4^ pfu/well), X-axis, followed by a series of two-fold dilutions down to a lowest multiplicity infection of 3.9x10^-4^ for vaccinia virus, and 9.76x10^-4^ for variola, cowpox and monkeypox viruses. The Y-axis corresponds to percent of infected cells. Incubation times ranged from 5–28 hours post infection (hpi). NC = Negative control.

Vero E6 cells were plated in growth medium in black 96-well, clear bottom plates (Perkin Elmer, Whaltham, MA, USA) at 1.7x10^4^ cells per well and were allowed to attach overnight at 37°C, 5% CO_2_. On the day of infection viral stocks were sonicated three times for one minute with 20-second incubations on ice between sonications and pre-diluted 100-fold, using 2% FBS infection medium. Further viral dilutions were prepared in dilution plates and then transferred onto test plates. At the infection step, growth medium was removed from test plates and replaced with 50uL/well of virus. Infected cells were incubated at 37°C, 5% CO_2_. For experiments with variola virus, the incubation temperature was 35.5°C. In order to find optimal assay conditions we evaluated growth dynamics for each virus, and prepared multiple plates to test different incubation times. Infectivity assays were established using vaccinia Wyeth virus, which was prepared in a series of nine two-fold dilutions, starting from an intended MOI = 1 (1.7x10^4^ pfu/well), followed by MOIs of 0.5, 0.25, 0.125, etc. (Fig 1). Vero E6 cells were infected with 50uL per well of viral dilutions, in duplicates and incubated for 5, 7, 17 and 24 hours at 37°C. Each plate had 4–6 wells with infection medium only, that were used as uninfected control for data analysis. At the end of each incubation period, cells were fixed with formaldehyde and stained with anti-vaccinia virus polyclonal antibody.

### Plaque Assay

Plaque assay was used to determine viral titers for the stocks used during assay development and method comparison experiments. Vero E6 cells were plated in 6-well dishes (Corning, Tewksbury, Mass) at 1.4x10^6^ cells per well and incubated overnight at 37°C, 5% CO_2_. Virus samples were sonicated three times at 160 Watts for one minute with 20-second incubations on ice between sonications. Ten-fold dilutions were prepared for each viral stock from 10^−3^ to 10^−8^. Growth medium was replaced with 1mL per well of virus suspension at each different dilution, in duplicates. Infected plates were incubated at 37°C, 5% CO_2_ for one hour, and then, viral inoculum was replaced with 2% FBS infection medium. Plates with infected cells were incubated at 37°C, 5% CO_2_ for 48 hours. Cells infected with variola virus were incubated at 35.5°C, 5% CO_2_ for 96 hours. Monolayers were subsequently fixed and stained with crystal violet containing 10% formalin. Plaques were counted and concentrations for each stock were calculated using the following formula:

$$\frac{Mean\;number\;of\;plaques}{Inoculum\;per\;well\;\left(mL\right)\times Dilution\;factor\;for\;the\;well}$$

### Concordance between HC-IA and plaque assay

For comparison and correlation between HC-IA and traditional plaque assays, we used 13 vaccinia virus stocks. For HC-IA, a viral stock with known titer from previously performed plaque assay was used as “reference” starting at a multiplicity of infection (MOI) ranging from 0.3–0.6. Viral stocks with unknown titers were treated as “samples” and were initially run at a 1:1500 starting dilution. When required, viral stocks with low titers were re-tested at a 1:200 or 1:1000 starting dilution, followed by a series of two-fold dilutions. For the comparison with plaque assay, all viral stocks were tested following the procedure described above.

### Development of Staining Procedure with 5% MicroChem-Plus inactivation treatment

Variola virus infectivity and neutralization experiments were performed in a BSL- 4 laboratory and experimental plates were required to be disinfected before transporting to BSL- 2 laboratory for data acquisition. In compliance with an Environment, Safety, and Health Compliance Office (ESCHO) approved protocol, 5% MicroChem-Plus (National Chemical Laboratories Inc, Philadelphia, PA, USA) was used to disinfect all material from the BSL- 4 before being removed from the laboratory. We evaluated the effect of MicroChem-Plus treatment on immunostaining by comparing fluorescent intensity between different staining conditions. Staining conditions with MicroChem-Plus treatment were optimized in the BSL- 2 laboratory using vaccinia virus. Following the developed infectivity assay protocol (HC-IA), Vero E6 cells were infected at MOI 0.25 and incubated for 17 hours. After fixation with 4% formaldehyde, wells in 96-wellplate were divided into four groups depending on the order cells were treated with 5% MicroChem-Plus, and included the following: “No MicroChem-Plus” which was used as a control condition, “After fixation” (MicroChem-Plus added post fixation step), “After permeabilization” (MicroChem-Plus added after permeabilization step), and “After secondary antibody” (MicroChem-Plus added after last step of staining protocol). MicroChem-Plus treatment lasted 5 minutes, and was followed with 5 washes with PBS.

### High Content Orthopoxvirus Neutralization Assay (HC-OVNA)

Vero E6 cells were plated in growth medium in black 96-well, clear bottom plates at 1.7x10^4^ cells per well and were used the following day. During assay development VIG was used to assess neutralization of tested Orthopoxviruses. VIG and Orthopoxviruses were prepared at 2X concentration and then mixed together in equal volumes, followed by a 30 minute pre-incubation at 37°C. At the end of pre-incubation, growth medium from the assay plate was replaced with pre-incubated mixture of virus and VIG and incubated for 17 hours at 37°C, 5% CO_2_. VIG was prepared at three-fold serial dilutions, starting at 1:25 dilution (2X) and mixed with the virus at MOI 0.5 (2X). Every test plate had four medium only and virus only wells that were used as non-infected and infected controls, respectively. After a 17 hours incubation, cells were fixed, permeabilized, and stained with anti-vaccinia antibody for virus detection, as described in HC-IA, with the addition of the 5% MicroChem-Plus treatment step for the variola virus based assay. According to the protocol, microtiter plates containing formaldehyde-fixed variola virus were submerged into 5% MicroChem-Plus solution for 5 minutes and then were transferred to BSL-2 laboratory. MicroChem-Plus solution was removed and plates were washed five times with PBS. Results were acquired on ArrayScan and analyzed using Columbus software and reported as percent infected cells per well. The dilution at which 50% of cells are neutralized was calculated in GraphPad Prizm, using a sigmoidal dose-response nonlinear regression algorithm.

### Data Acquisition and Analysis

Image analysis was performed using Columbus Scope software, where all cells were identified first by nuclear (DAPI) and cytoplasmic staining (HCS Cell Mask Deep Red Stain). Cells infected with virus were identified with rabbit anti-vaccinia virus antibody, following by fluorescently labeled anti-rabbit antibody. The numbers of infected and non-infected cells were scored and the percent of infected cells was calculated by dividing the number of “infected” cells by the number of “total cells” identified in the well and multiplying the resulted ratio by 100%.

During virus titration experiments, we observed that the percent of infected cells directly correlated with viral concentration. The highest percent of infected cells was detected in the wells that had high concentrations of virus and detection of infected cells gradually decreased with each additional virus dilution step. For the viral stocks with known starting titers (pfu/mL) we calculated an expected viral concentration for each dilution point in a dilution series and then correlated it to corresponding percent of infected cells. We employed GraphPad Prizm software, version 5 (La Jolla, CA, USA) to graph the results of virus infection, where estimated virus titers (pfu/mL) in a log-transformed dilution series were positioned on the X-axis and percent of infected cells were on the Y-axis. Data was analyzed using non-linear regression sigmoidal dose-response variable slope algorithm. Since viral stocks that were used for infection dynamics experiments had known starting titers, calculated infectious concentrations 50% (IC_50_) were reported in pfu/mL and MOI.

In the high-content infectivity assay, we used Parallel Line Analysis Software PLA 2.0 (Stegmann Systems, Rodgau, Germany) to calculate *relative* titers (pfu/mL) for samples with unknown potency from reference stocks with known pfu/mL concentrations that were previously determined by plaque assay. Log-transformed dilution series for reference stocks and samples were plotted on the X-axis and corresponding percent of infected cells on the Y-axis. For analysis, we used four-parameter logistic curve and calculated dilution at IC_50_ for samples and reference standard. The relative potency is the exponentiated distance between IC_50_ value of the reference stock and IC_50_ value of the sample. Since reference stock had a known starting concentration and the reference standard is similar to the sample, the relative potency was used as a factor to estimate the potency of the sample. For the high-content Orthopoxvirus neutralization assay, the half maximum effective concentration (EC_50_) values were calculated using GraphPad Prizm software. Percent of infected cells (y-axis) were plotted against serum dilutions (x-axis) generating a sigmoidal curve. Serum dilutions that resulted in 50% neutralization of viral infection were calculated using non-linear regression sigmoidal dose-response (variable slope) algorithm.

To evaluate assay reproducibility, we applied Z-factor statistical method, which is commonly used to estimate reproducibility and robustness of screening assays. This parameter assesses, in part, assay quality by calculating separation between positive and negative signals. Calculated Z-factor values between 1 and 0.5 refer to an excellent assay, while values between 0.5 and 0 indicate a marginal assay. Z-factor was calculated using the following formula [10]:

$$Z=1-\frac{3\;\times \left(SD\;Infected+\;SD\;Neutralized\right)}{\left(Mean\;Infected-\;Mean\;Neutralized\right)}$$

In concordance evaluation between HC-OVNA and HCS-GFP assays, results from HCS-GFP neutralization assay were analyzed in GraphPad Prizm using a variable slope sigmoidal equation, as previously described [6]. Calculated inhibitory concentration 50 (ID_50_) that neutralizes 50% of vaccinia virus Western Reserve GFP expressing virus in HCS-GFP assay was compared to EC_50_ values from HC-OVNA. Results from both assays were compared for each sample and analyzed in GraphPad Prizm using correlation algorithm with 95% confidence interval in two-tailed test.

To evaluate the agreement between plaque assay and HC-IA we used GraphPad Prizm correlation algorithm with 95% confidence interval in two-tailed test and compared titers from plaque assay to relative potency values from HC-IA.

### Evaluation of serum neutralizing activity from monkeypox virus challenged prairie dogs

Serum samples from prairie dogs (PDs) infected with monkeypox virus study were kindly provided by Christina Hutson [11]. Collected serum samples were from two prairie dogs intranasally challenged with West African monkeypox virus (strain MPXV-USA-2003-044, collected during the 2003 U.S. outbreak) at 5 x 10^3^ pfu and included sera collected on days 3, 6, 13, 20, 24 post-challenge and on the day 31 when they were euthanized. We evaluated virus neutralization using vaccinia virus Wyeth strain, where prairie dog serum samples were tested at titration ranging from 1:40 to 1:40960, in a series of four-fold dilutions. As per the developed HC-OVNA protocol, equal volumes of vaccinia virus were mixed with titration of PD serum and pre-incubated for 30 minutes at 37°C, followed by 17 hours infection of Vero cells.

### Correlation between HC-OVNA and HCS-GFP

The HCS-GFP Neutralization Assay has been previously described [6] and is based on recombinant vaccinia virus Western Reserve virus expressing green-fluorescent protein (WR-GFP). Agreement between HC-OVNA and HCS-GFP assays was assessed with 49 human serum samples from the study “Evaluation of the Serologic Diagnostics for DryVax Smallpox Vaccine”, collected according to CDC Institutional Review Board protocol 3349. Available serum samples were divided into four categories: Category “A” consisted of serum collected from participants that were vaccinated with smallpox vaccine for the first time at adulthood; Category “B” includes serum from individuals that were vaccinated at adulthood and were boosted 3 years after first vaccination; Category “C” included individuals previously vaccinated at childhood (30 + years) and boosted once in adulthood; Category “D” included serum from individuals that were vaccinated in childhood and boosted multiple times in adulthood (Table 1). For each category, the following time points were selected: day 0, day 7, day 21 and 6 months post vaccination with DryVax vaccine. Each serum sample was tested on three separate days by each assay. Samples were tested in four-fold serial dilutions ranging from 1:40 to 1:40960 and were prepared according to each assay procedure. Results for each assays were acquired on ArrayScan. Collected images for HC-OVNA were analyzed using Columbus Scope algorithm and then plotted using GraphPad Prism 5.0 software. Images from HCS-GFP assay were analyzed using Target activation, version 3 algorithm from ArrayScan, followed by analysis using GraphPad Prizm [6]. For correlation analysis EC_50_ values from HC-OVNA were plotted against ID_50_ values from HCS-GFP assay and analyzed in GraphPad Prizm.

**Table 1:** Four categories of human serum samples selected for the study.

Vaccination status	Childhood	Adult 1	Adult 2	Adult 3+
Adult only (“A”); n = 3	-	X*	-	-
Adult primary and boosted 3 years (“B”); n = 3	-	X	X*	-
Childhood primary and boosted once at adulthood (“C”); n = 5	X	X*	-	-
Childhood primary and multiple boosts at adulthood (“D”); n = 7 ^ª^	X	X	X*	X*

^ª^ Results include samples from adult boost vaccinations collected at different times for the same individuals.

X*—Indicates serum samples collected at selected vaccination time.

X—Indicates previous vaccination times.

## Results

Infectivity assays were established using vaccinia Wyeth virus. The fluorescent signal associated with vaccinia virus infection at MOI 1 was reproducibly detectable at 5 and 7 hours post infection (hpi) and corresponded to 15% and 40% of cells infected, respectively (Fig 1). Infections at MOIs of 0.5, 0.25 and lower showed significantly less fluorescent signal at early time points. An MOI of 0.5 corresponded to 3% and 18% of infected cells for 5hpi and 7hpi, respectively, resulting in a low dynamic range. However, infections at 17 and 24 hours showed that percent of infected cells increased up to 95% at MOI 1, 0.5, and 0.25. Based on these results we selected 17 hours incubation time for vaccinia virus infection experiments, which allowed us to have a rapid high-throughput assay.

Time course experiments were performed with similar dilution ranges for variola, cowpox and monkeypox viruses. Infection dynamics for these viruses are slower than we observed with vaccinia virus and required longer incubation times, ranging between 24–28 hours in order to reach a minimum of 90% of infected cells per well (Table 2). For vaccinia virus the infectious concentration 50 (IC_50_) values at 17 hours post infection were 1.65x10^4^ pfu/mL, that corresponded to MOI 0.055, while for cowpox virus the IC_50_ value was 1.57x10^5^pfu/ml (MOI 0.53). By 24hpi all tested Orthopoxviruses had a percent of infected cells above 90% for the first dilution point (an intended MOI 1) with 3 to 5 fold difference in IC_50_ values. Still, vaccinia virus continues to have the fastest infection dynamics (IC_50_−1.01x10^4^ pfu/mL, or MOI 0.035), while monkeypox virus infection was the slowest (IC_50_−4.75 x10^4^ pfu/mL, MOI 0.158). Cowpox and variola viruses had somewhat similar infection dynamics with IC_50_−2.55 x 10^4^ pfu/mL (MOI 0.095) and 3.25 x10^4^ pfu/mL (MOI 0.108) respectively. As a result we selected 24 hours incubation time for infections with variola, monkeypox and cowpox viruses.

**Table 2:** IC_50_ infectivity values for tested Orthopoxviruses.

HPI	Vaccinia		Cowpox		Monkeypox		Variola	
	pfu/mL	MOI	pfu/mL	MOI	pfu/mL	MOI	pfu/mL	MOI
**17**	1.65x10^4^	0.055	1.57x10^5^	0.53	--	--	--	--
**20**	--	--	--	--	--	--	7.87x10^4^	0.261
**24**	1.01x10^4^	0.035	2.55x10^4^	0.095	4.75x10^4^	0.158	3.25x10^4^	0.108
**28**	--	--	--	--	1.61x10^4^	0.054	5.66x10^3^	0.019

**Fig 2:**
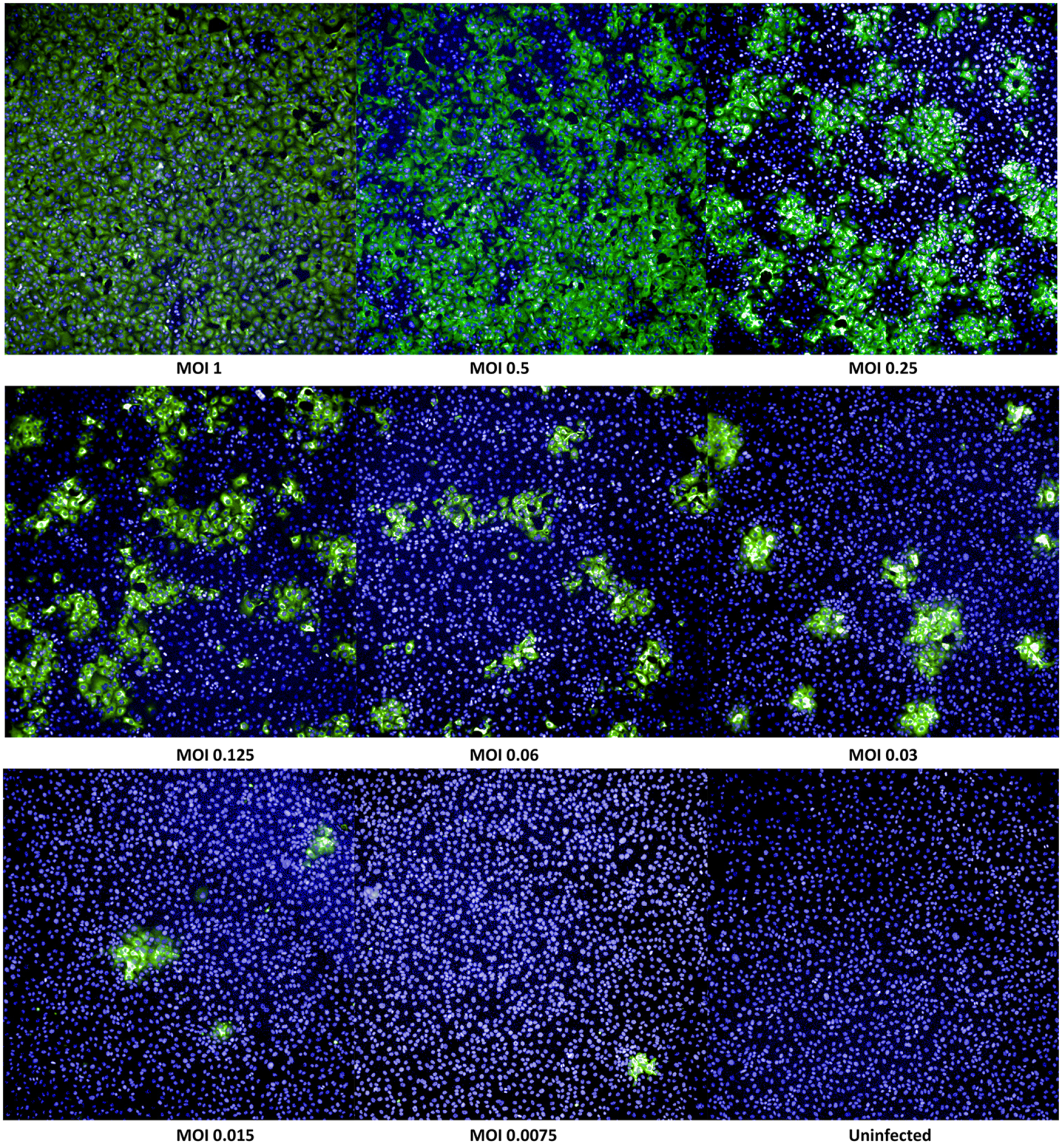
Variola virus infection in Vero E6 cells at different MOI at 24 hours post infection. Cell images were collected at 5X magnification with 9 fields per well. Variola virus (Solaimen) was titrated at different MOI concentrations. Wells with uninfected control cells were included on each plate to be used for gating purposes during image analysis. Cells were identified by nuclear staining (pseudo-colored blue) and virus presence was detected with indirect staining (pseudo-colored green). Images show infected cells in one field per well at different MOI after 24 hours infection.

Fig 2 represents variola virus infection at different MOI concentrations, detected at 24 hours post infection. The number of infected cells is the highest at MOI 1 and gradually decreasing with virus titrations, allowing to identify an individual foci.

### Development of Staining Procedure with 5% MicroChem-Plus treatment

**Fig 3:**
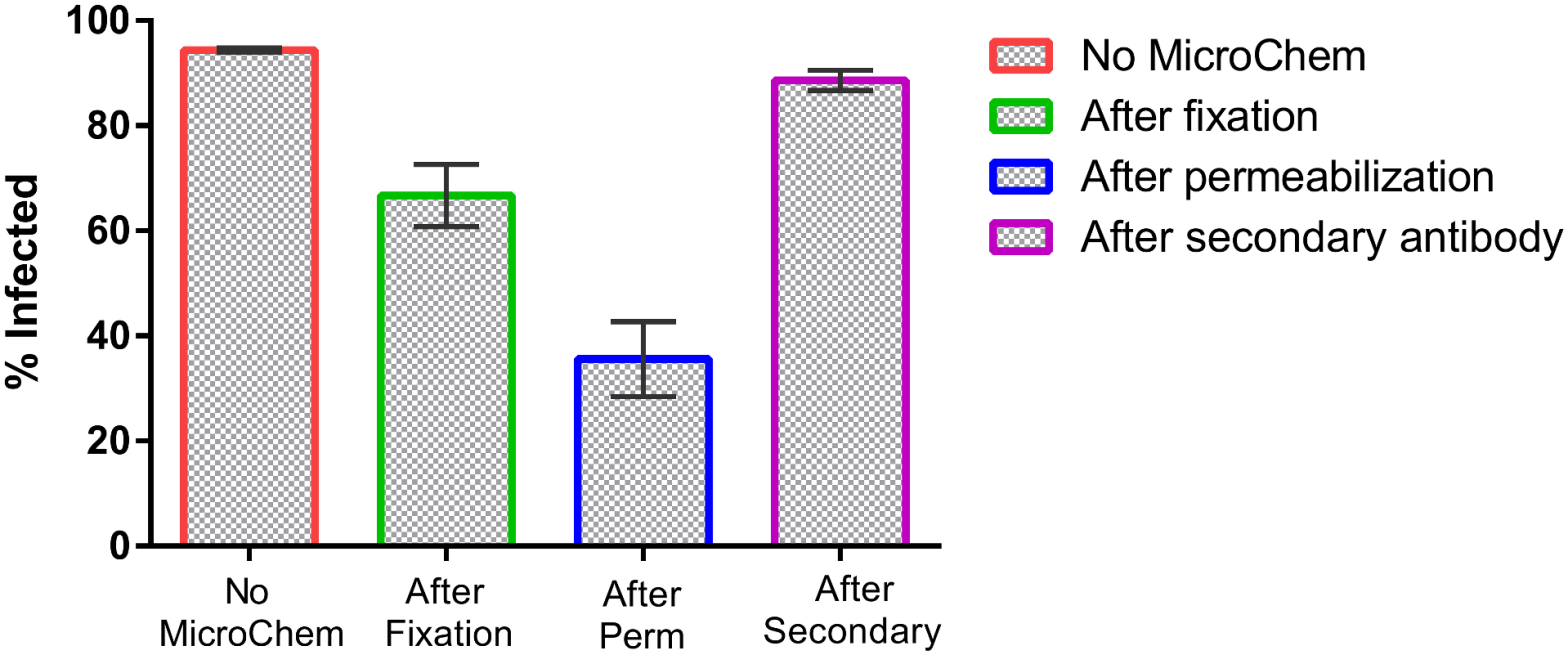
MicroChem treatment experiment. Treatment of cells with disinfectant after completion of staining procedure produced similar signal intensity as the non-treated control. The Y-axis shows the percent of infected cells for each condition tested. The list of different conditions is shown on X-axis. MicroChem treatment at the end of staining procedure produced comparable signal as control (No MicroChem).

To evaluate the effect of MicroChem-Plus disinfecting reagent on signal intensity from the virus stained with anti-vaccinia polyclonal detection antibody, 5% solution of MicroChem-Plus was applied at different steps of staining protocol. MicroChem-Plus treatment had a minimum effect on vaccinia virus signal intensity when incorporated at the end of the staining protocol. Signal from the wells that received MicroChem-Plus was comparable to the signal of control wells (Fig 3). However, signal intensity decreased when MicroChem-Plus was introduced after fixation or permeabilization step. Due to the drop in signal intensity when MicroChem-Plus treatment occurred prior to staining, all immunostaining steps for experiments with variola virus were conducted within the BSL- 4 and MicroChem-Plus treatment occurred at the end of the staining procedure.

### Correlation study between HC-IA and Plaque Assay

**Fig 4:**
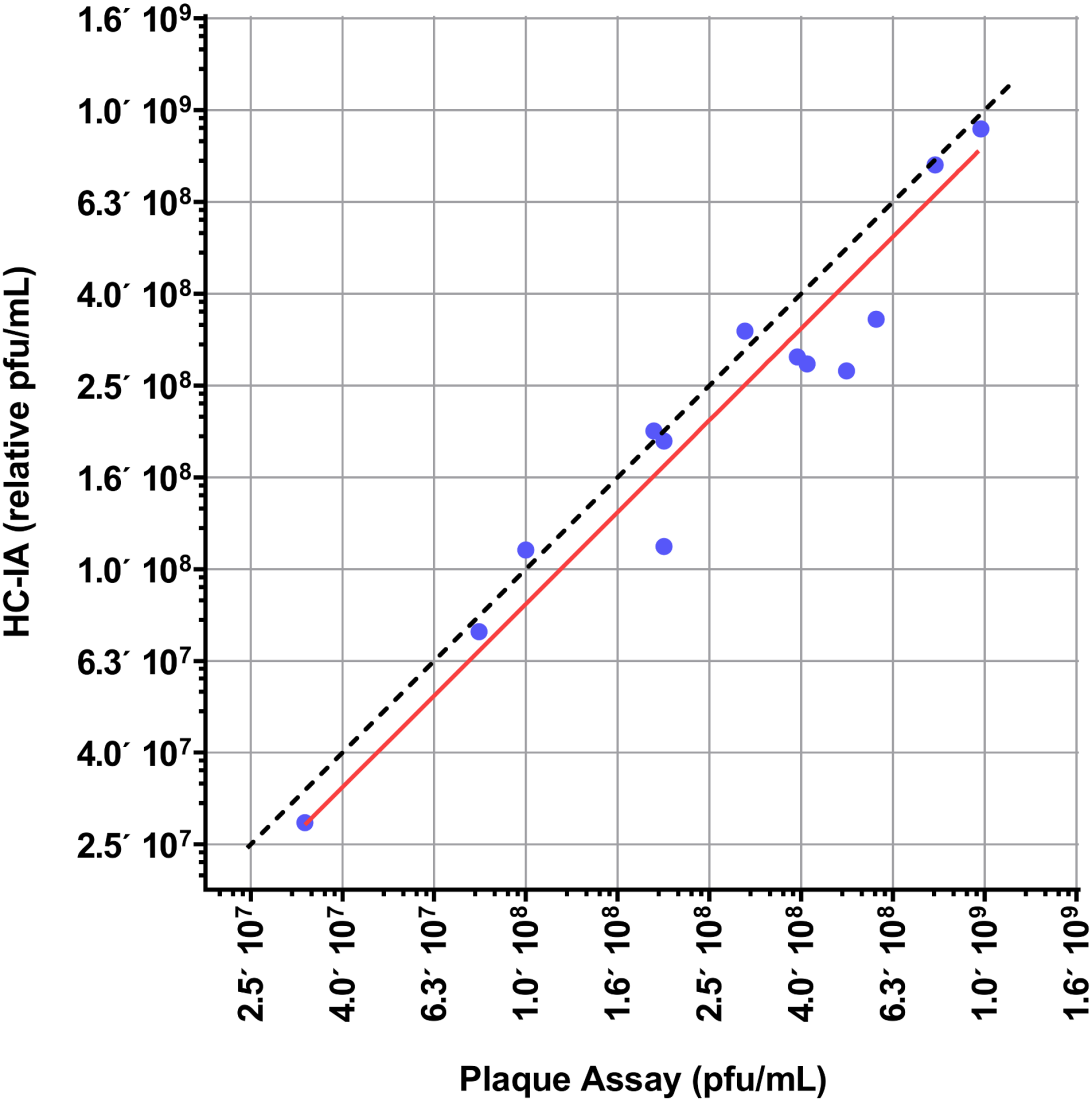
Assay Concordance plot. Bivariate scatter correlation plot between vaccinia virus infectivity measured by high-content analysis based infectivity assay (HC-IA) (Y-axis) and the plaque assay (X-axis) presented in log 10 scale. Potencies of thirteen stocks of vaccinia virus were measured in plaque assay and HC-IA. Results were analyzed using GraphPad Prizm software showing significant correlation between image-based and standard plaque counting assays (Pearson r—0.95, P value—0.0001, R^2^—0.9, slope 0.83). Dashed line represents the ideal linear curve with slope of 1, while solid line is an observed concordance curve.

We evaluated the correlation between the HC-IA and standard plaque assay using vaccinia Western Reserve and vaccinia Wyeth virus strains (Fig 4). Thirteen vaccinia virus stocks were tested in duplicate with both assays. The majority of viral stocks had titers between 10^8^−10^9^ pfu/mL and were tested in high content infectivity assays starting at a 1:1000 dilution, followed by two-fold serial dilutions. With these starting dilutions, a few low titer viral stocks infected less than 50% of cells per well, instead of the targeted 90–95%, after 17 hours of incubation. These stocks were re-tested with starting dilution at 1:200, followed by eleven 2-fold dilutions, which resulted in 90–95% of cells per well detected as infected at the lowest dilution tested. For future experiments we decided to follow the rule that if stock is expected to have potency at 10^8^−10^9^ pfu/mL, then the starting dilution should be at 1:1000, but for any stocks with lower or unknown potency, a starting dilution at 1:200 dilution, followed by eleven 2-fold dilutions was used.

The results from the HC-IA method were reported as percent of infected cells per well. In order to calculate the relative potency (pfu/mL) of viral stocks with the HC-IA, PLA software was utilized. Selected reference stock sample had a known viral titer (pfu/mL) previously determined by plaque assay. The HC-IA method results were plotted and analyzed with four-parameter logistic fit algorithm. The IC_50_ dilution values in the HC-IA for the reference and sample stocks were calculated and used to estimate the relative potency (pfu/ml) of the sample by measuring the change in interpolated dilutions at IC_50_ between reference and sample stocks. To estimate the agreement in the results for each sample between the new method and plaque assay, calculated relative potency of a sample from HC-IA was compared to a titer value from plaque assay (Fig 4). The results of correlation experiment showed that there is a strong agreement between HC-IA and plaque assay (Pearson r-0.95, R^2^-0.9, Slope-0.83). These results demonstrate that HC-IA measures vaccinia virus infectivity with the similar sensitivity as plaque assay.

### High Content Orthopoxvirus Neutralization Assay

**Fig 5:**
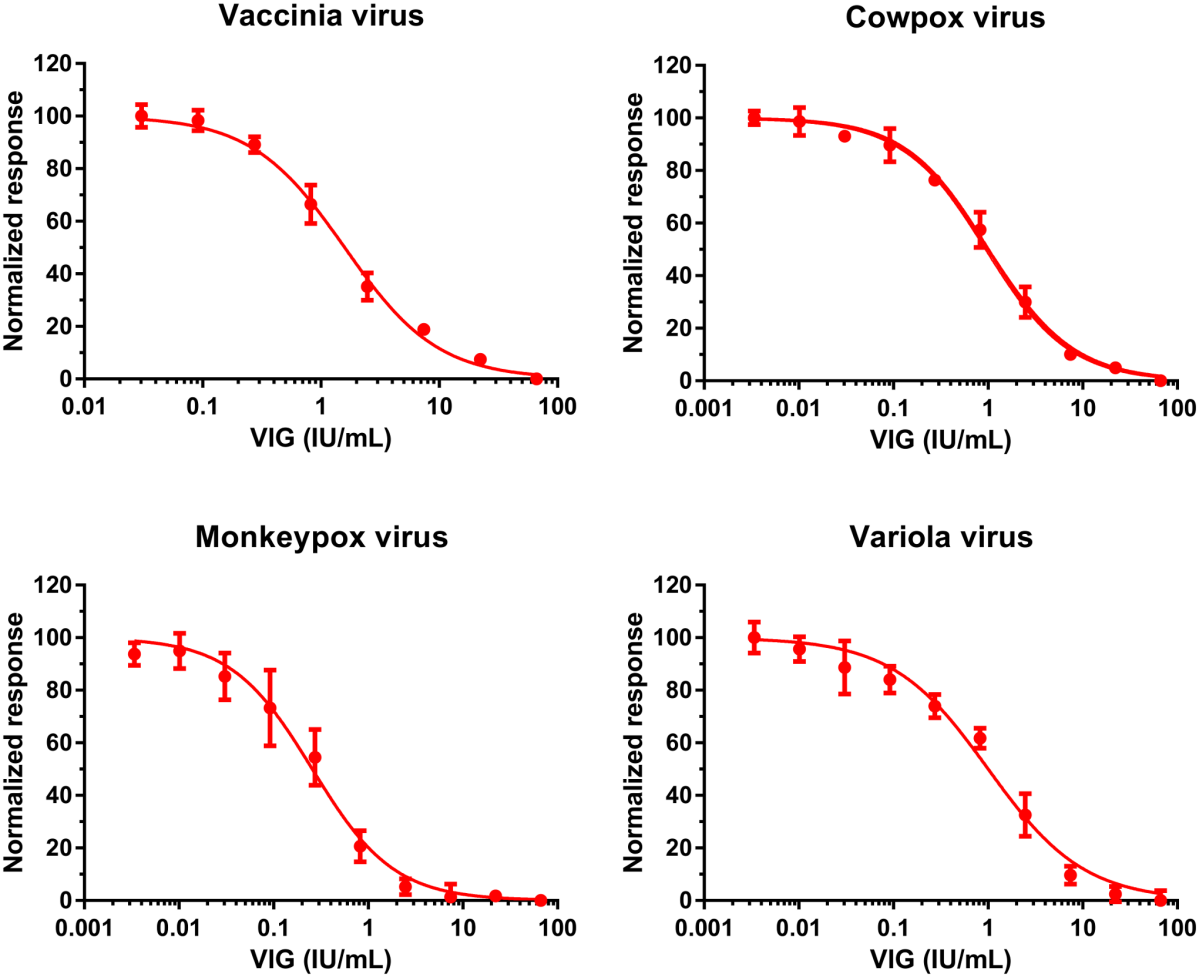
Neutralization of viral infection with VIG. Vaccinia Immune Globulin was titrated in 3-fold serial dilutions starting from 1:50 dilution (66.67 IU/mL) and was pre-incubated for 30 minutes at 37°C with test virus (cowpox, vaccinia or monkeypox viruses), or at 35.5°C for variola virus. Pre-incubated mixture was transferred to Vero E6 cells and incubated for 17 hours (vaccinia virus), or 24 hours (cowpox, monkeypox and variola viruses).

Using vaccinia Wyeth virus as the target of neutralization, we prototyped development of the high content neutralization assay (Fig 5). Based on the results from vaccinia virus infectivity assay, we selected 17 hours incubation post infection with a viral MOI 0.25 that expected to produce infection in approximately 90% of cells in the absence of any neutralizing serum. VIG neutralized vaccinia virus infection at EC_50_ of 1.59 IU/mL, or at 1:2100 fold dilution of the product. Similar neutralization experiments were conducted with monkeypox, cowpox, and variola viruses, where cells were infected at MOI 0.25 and neutralized with titration of VIG, but allowed to incubate for 24 hours post infection. Since infection dynamics experiment (Fig 1) showed that monkeypox, cowpox and variola viruses grew at a slower rate than vaccinia virus, we selected a longer incubation time, but kept the same MOI concentration. Cowpox and variola virus infections were 50% neutralized at VIG concentrations 0.9 IU/mL (1:3311 fold dilution) and 1.0 IU/mL (1:3295 fold dilution), respectively. Monkeypox virus infection was neutralized at 0.3 IU/mL (1:12603 fold dilutions). The differences in VIG EC_50_ concentrations are directly associated with observed infection dynamics for all tested viruses. According to the results of monkeypox virus infection dynamics (Fig 1), infection at MOI 0.25 for 24 hours produces approximately 65% infection positive cells, whereas similar conditions for cowpox and variola viruses correspond to approximately 80–85% infected cells, which require higher amounts of VIG for virus neutralization.

### Z-factor

**Fig 6:**
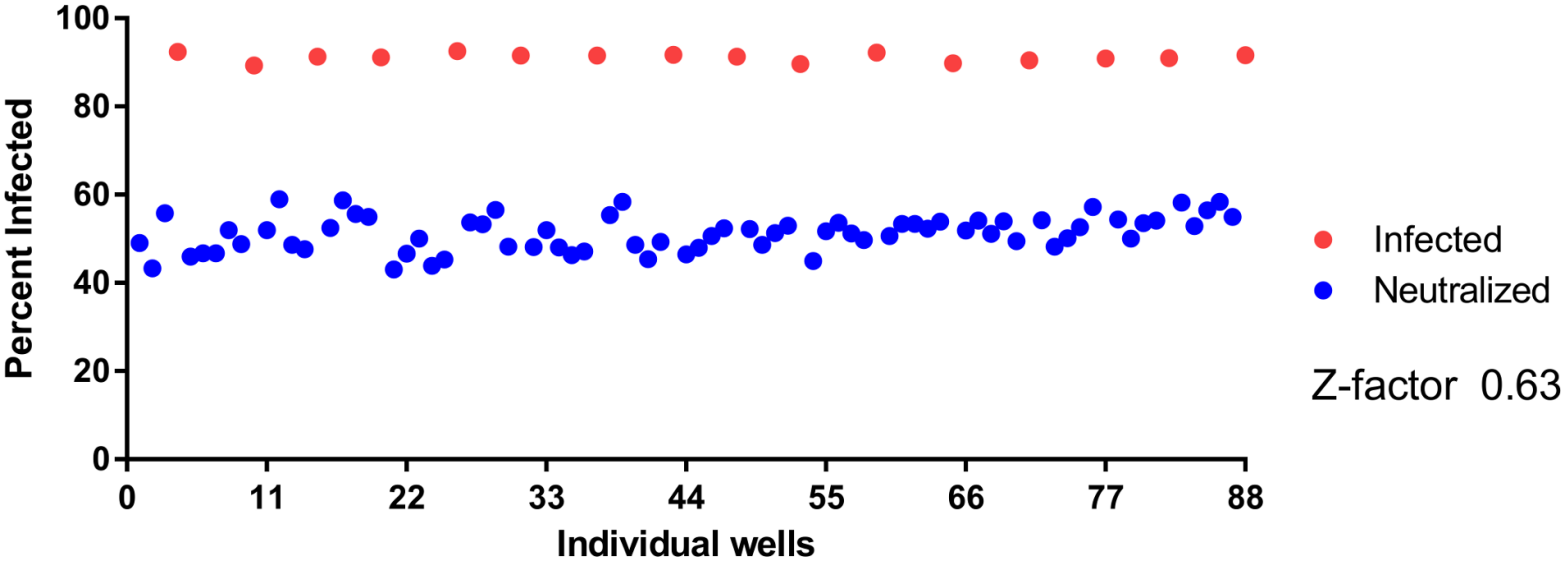
Assay reproducibility. Z-factor experiment was performed to evaluate assay reproducibility, where 20% of a plate had wells infected with vaccinia virus at MOI 0.250, and the rest of the plate had wells that were infected (MOI 0.250) and neutralized with pooled human serum at 1:100 dilution Calculated Z-factor value is 0.63 indicating that the developed assay is robust and reproducible.

To evaluate assay reproducibility we applied Z-factor statistical method (Fig 6). Vero cells were either treated with vaccinia virus at MOI 0.250 (“Infected”), or a mixture of virus and pooled human serum from previously immunized individuals at 1:100 dilution (“Neutralized”). Mean and standard deviations of “Infected” and “Neutralized” wells were calculated and used to determine Z-factor value. According to the results, the calculated Z-factor is 0.63, which falls within the range for a robust and reproducible assay.

### Correlation between HC-OVNA and HCS-GFP

**Fig 7:**
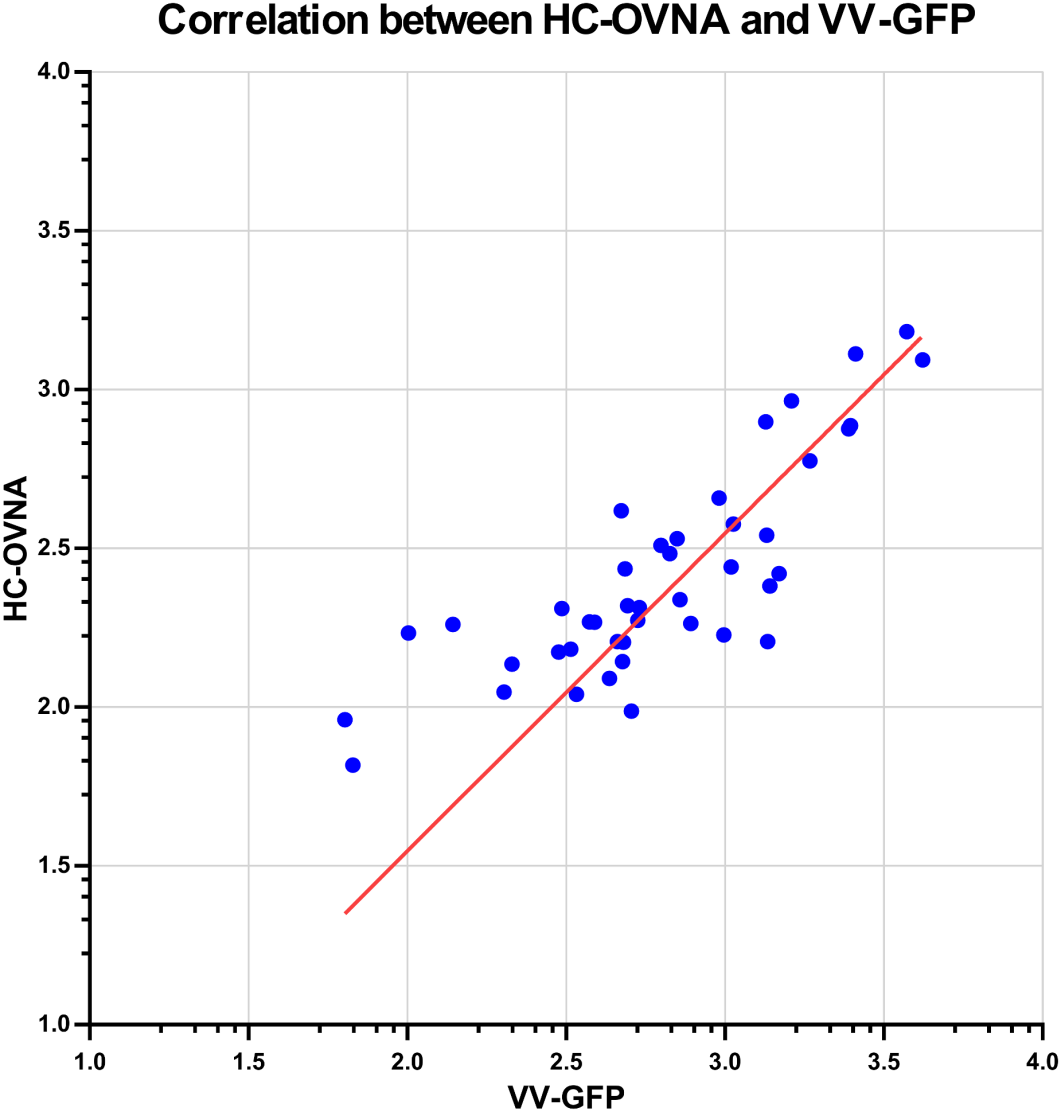
Bivariate scatter correlation plot between two imaging assays. New Orthopoxvirus neutralization assay (HC-OVNA), Y-axis, and GFP-expressing vaccinia virus (HCS-GFP), X-axis, presented in log 10 scale. Correlation between two assays was evaluated using 49 human serum samples from smallpox vaccine study (CDC IRB protocol 3349). Mean EC_50_ values from neutralization curves from HC-OVNA and HCS-GFP assays were plotted against each other (log scale) and analyzed using GraphPad Prizm software, showing significant correlation between two assays (Pearson r—0.9, P value—0.0001, R2–0.8).

The mean EC_50_ values of serum titrations for three runs were collected for both assays and were plotted against each other (Fig 7). Samples that did not neutralize viral infection (Category A and C days 0, day 7) at 1:40 dilution were excluded from correlation analysis. The correlation analysis between HCS-GFP and HC-OVNA was performed using ID_50_ values (HCS-GFP) and EC_50_ results (HC-OVNA) for 43 samples and appears to be significant with correlation coefficient = 0.9, and coefficient of determination = 0.8 (p value < 0.0001).

**Fig 8:**
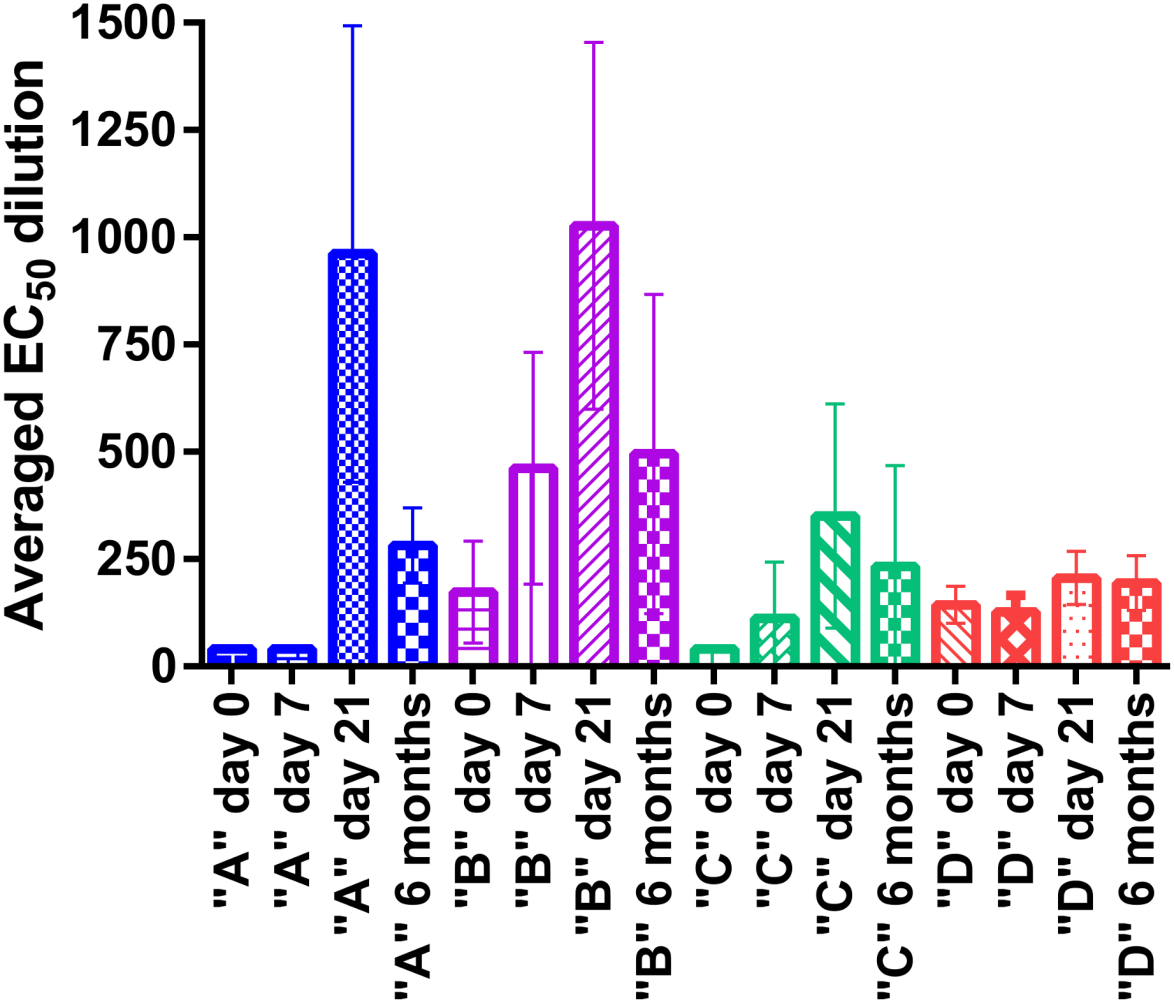
Average immune response for different vaccinee categories. Neutralization responses for each vaccinee category were grouped according to blood draw days. Calculated average and standard deviation values were based on the results from several vaccinee per each category.

To further evaluate assay performance we used HC-OVNA derived EC_50_ neutralizing dilution values from correlation experiment and compared the changes in levels of serum neutralizing antibodies for different vaccinee categories. (Fig 8). The EC_50_ dilution results were grouped by vaccination status (categories A, B, C, D) and then by serum collection days. The EC_50_ results for each category were averaged and analyzed for days 0, 7, 21 and 6 months. For the first time vaccinee (Category A) we were expecting to see a significant increase in neutralizing antibodies by day 21 and decrease in antibody levels by 6 months [12, 13]. Consistent with our expectations the highest level of IgG response was observed on day 21, with EC_50_ at 1:960 serum dilutions (median 1241, range 348–1295). Neutralization responses from serum samples collected on days 0 and 7 were rated as non-neutralizing with EC_50_ values below a 1:40 dilution, since minimal to no neutralization activity was observed. By 6 months the amount of serum neutralizing antibodies was reduced, resulting in EC_50_ dilutions at 1:280 dilution (median 263, range 206–377).

Category “B” included serum samples from the same individuals as in Category “A” that were re-vaccinated three years after first smallpox vaccination. We observed that the remaining neutralizing antibodies present in serum on day 0 of re-vaccination were slightly lower compared to the amount of neutralizing antibodies present in serum 6 months after primary vaccination and almost 75% lower than on day 21 (category “A”). The increase in immune response for category “B” was observed by day 7 and reached its peak on day 21 with EC_50_ dilution at 1027 (median 791, range 770–1521) and lowered almost 2-fold by 6 month (mean 495, median 323, range 241–922).

For Category “C” (re-vaccination after 30 or more years post primary vaccination) neutralizing response, from day 0 serum samples were rated as non-neutralizing with EC_50_ dilution values below a 1:40. By day 7 a discernable neutralizing antibody titer (mean 112, median 97, range 40–105) was measured, and reached its peak at day 21 (mean 290, median 191, range 113–596). By six months the amount of neutralizing antibodies has dropped slightly with mean EC_50_ dilution at 270 (median 254, range 118–456).

The EC_50_ results of the immune response for individuals that were vaccinated multiple times (category “D”) was relatively similar between all serum collection days, with a slight increase in potency for day 21 serum samples. At day zero, the mean EC_50_ dilution was 160 (median 169, range 112–185) and slightly increased by day 21 (EC_50_ mean 215, median 204, range 160–340), and stayed unchanged for six months (EC_50_ mean 210, median 218, range 110–277). It appears that with repeated vaccinations (category “D”) the EC_50_ dilution values are lower than with initial vaccination or one-time three-year boost, and do not significantly change depending on the day serum was collected.

### Screening immune-response to immunization in prairie dogs

**Fig 9:**
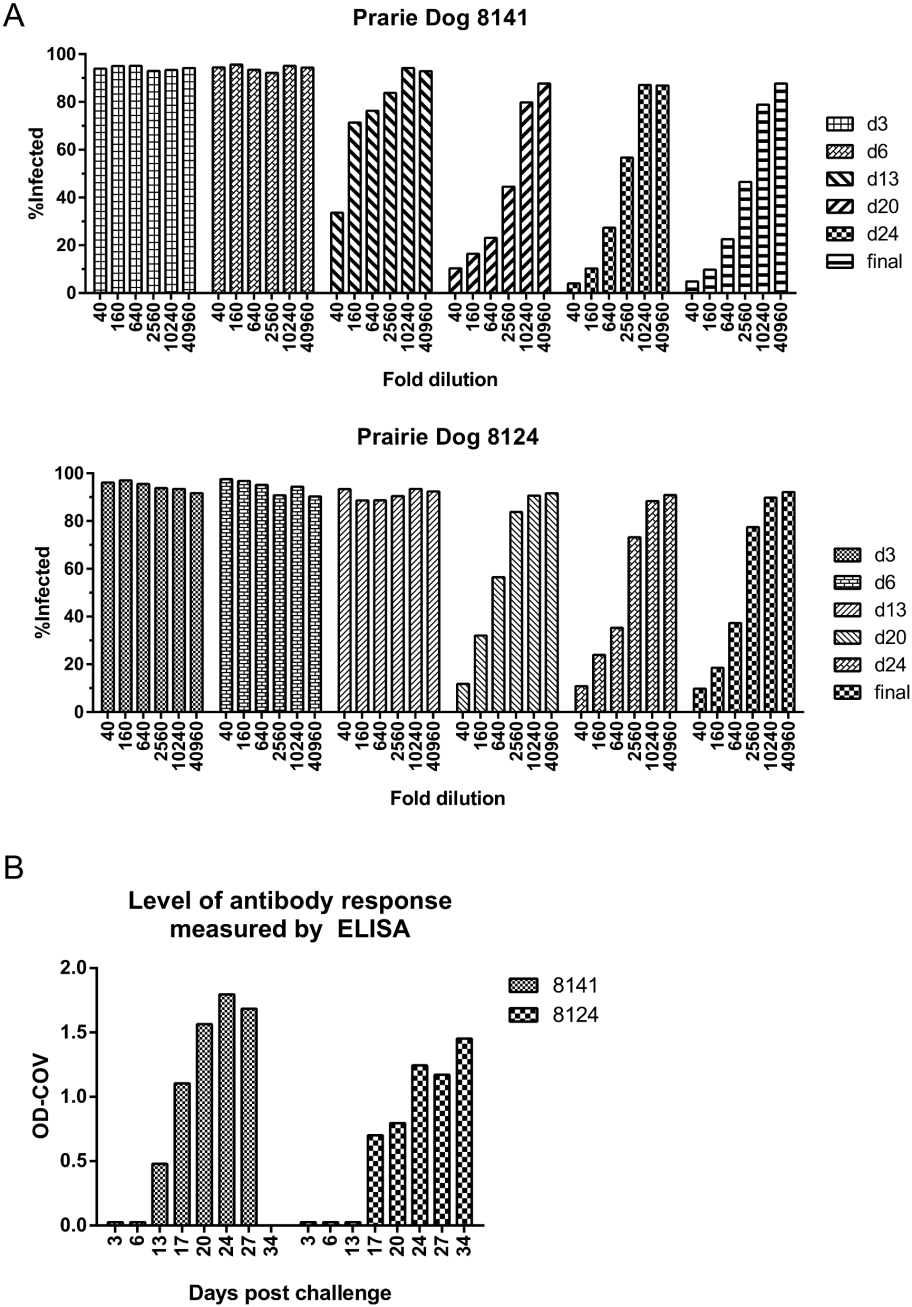
Screening of immune response to monkeypox virus challenge in prairie dogs. **A.** Serum samples from the monkeypox virus challenge study in prairie dogs were tested in high-content Orthopoxvirus neutralization assay at four-fold serial dilutions, starting from 1:40 dilution. **B.** Evaluation of antibody production in prairie dogs post monkeypox virus challenge using ELISA.

In this experiment we compared the results of the functional immune response measured by HC-OVNA to the total amount of anti-Orthopoxvirus IgG antibodies measured by ELISA. Serum samples from two prairie dogs intranasally challenged with monkeypox virus (strain MPXV-USA-2003-044 at 5 x10^3^ pfu) were tested in high-content Orthopoxvirus neutralization assay using Wyeth vaccinia virus. Serum samples were collected on days 3, 6, 13, 20, 24, and 31 post-challenge. No neutralization activity was observed on days 3 and 6 for PD 8141 (Fig 9A). However, the first signs of virus neutralization were detected from serum collected on day 13, followed by a gradual increase in virus inhibition from the samples collected on days 20, 24 and the day animal was euthanized (day 31). In PD 8124, neutralization of vaccinia virus infection was first detected from samples collected on day 20 and continuously increased until the day of euthanasia (day 31) (Fig 9A). The results of the neutralization assay were compared with previously performed ELISA data (Fig 9B) [11] and found two assays correlated. Similarly to HC-OVNA immune response to MPXV challenge was detected in ELISA on day 13 for PD 8141 and on day 20 for PD 8124 (day 17 serum samples were not available for HC-OVNA experiments). While ELISA evaluates the level of specific anti-viral IgG antibodies that bind, HC-OVNA is a functional assay that measures neutralizing activity of generated serum antibodies against tested Orthopoxviruses, that could be used as a complementary assay in virus challenge and vaccine studies.

## Discussion

Our objective was to develop new high-content, high-throughput infectivity and neutralization assays where Orthopoxvirus presence in a cell is fluorescently detected by a virus-specific antibody. We screened several commercially available anti-vaccinia polyclonal antibodies and found that they cross-react with variola virus, cowpox virus and monkeypox virus due to high genetic similarities found between members of the Orthopoxvirus genus. That allowed us to develop generic Orthopoxvirus neutralization and infectivity assays. However, the availability of Orthopoxvirus species-specific antibodies would allow adapting the infectivity assay into a specific diagnostic test.

In HCA assays, an infectious virus is detected per cell and its amount is reported as fluorescent intensity directly associated with antibody-stained virus. The advantage of HC-IA and HC-OVNA assays is the ability to detect the presence of an infectious virus significantly earlier than in plaque-based infectivity and neutralization assays. In imaging assays, vaccinia virus was detected 17 hours post infection, while in the plaque-based assay viral titers were measured after 48 hours of incubation.

Another advantage of these methods over plaque-based assays is the higher statistical significance of the collected results. During acquisition we gather data at 5X magnification, covering approximately 65% of the well, which corresponds to nearly 10,000 cells for each titration point in a series of eleven serial dilutions. Viral presence was detected per cell, at the early stages of foci formation. Collected results from a dilution series contribute to the calculations of IC_50_ and EC_50_ for infectivity and neutralization assays, respectively. At the same time, in plaque-based assays, viral titers were calculated based on the number of plaques found in the well corresponding to one dilution point. In the infectivity plaque assay viral dilutions that produced too many, or too few, to count plaques were excluded from the measurements. In addition to that, the working volumes of viral stocks and serum samples are significantly lower, than in plaque-based assays. In imaging assays we used 5 times less virus material than in plaque assay; and for virus neutralization experiments 5μL of serum was enough to start a dilution series. These low volume requirements not only alleviate the need to produce more viral stocks, but are also very important when serum sample quantities are small, especially when they come from miniature animals.

In the course of assay development we observed that four tested Orthopoxviruses have different infection dynamics when used at the same MOI concentrations. Consistent with prior observations, vaccinia virus grows the fastest among tested Orthopoxviruses and requires 17 hours infection to reach similar IC_50_ results as cowpox, variola, and monkeypox viruses with longer incubation times. At 24 hours post infection the IC_50_ values for vaccinia virus were almost five times lower than for monkeypox virus, making them the fastest and the slowest growing viruses, respectively. The developed infectivity assay was compared to plaque assay for estimation of viral stock potency. In our current version of the plaque assay, vaccinia virus grew in a medium that does not restrict viral spread, therefore, making incubation conditions for two assays somewhat similar. Comparison of viral potencies measured by both assays showed that two methods have a good agreement in the estimation of viral stock concentrations. However, HC-IA is a high-throughput test, which is less labor-intensive, and offers shorter turn-around time. Since commercially available vaccinia virus detection antibodies cross-react with other Orthopoxvirus species, the developed infectivity assay could be applied to measure viral stock concentrations of variola, cowpox, and monkeypox viruses, by adjusting incubation times that reflect each species growth dynamics.

Based on growth dynamics of vaccinia virus, for the development of neutralization assay, we decided to use MOI 0.25, which yields approximately 90% of cells infected at 17hpi. Applying the selected conditions for vaccinia virus infection neutralization experiment with titration of VIG resulted in a curve with more than 2-logs in dynamic range, which was used to estimate the EC_50_. We observed somewhat similar results when vaccinia virus neutralization conditions were applied to cowpox and variola viruses, but with longer incubation time (24 hours incubation). The EC_50_ values for VIG are very similar between the three viruses. However, selected infectivity conditions for monkeypox virus (MOI 0.25 for 24 hours) produced EC_50_ values lower than we observed for other tested viruses, which is expected, since above conditions should produce only 65% of infected cells according to growth dynamics experiments. We did not further optimize neutralization conditions for monkeypox virus infection, since this was not an objective of this study, but we believe that MOI 0.125 for 28 hours incubation would be optimal for virus neutralization with titration of VIG.

As a part of assay validation the comparison of the results from HC-OVNA with the results from HCS-GFP neutralization assay showed that two methods are very comparable, with high correlation coefficient value. To validate this assay further we evaluated the results of HC-OVNA for each vaccinee category and compared with available reports on changes in immune response after smallpox vaccination. Consistent with our earlier observations [13] for first time vaccinee the significant increase in immune response was observed by day 21. At six months the amount of neutralizing antibodies was almost 3 times lower than on day 21 post primary vaccination. It was previously reported that levels of neutralizing antibodies in primary vaccinee one year after vaccination were more than four-fold lower than on day 28 post-vaccination [12]. We saw that the remaining neutralizing antibodies present in serum on day 0 of re-vaccination (Category “B”–three year boost after primary vaccination) were slightly lower compared to the amount of neutralizing antibodies present in serum 6 months after primary vaccination and almost 75% lower than on day 21 post primary vaccination. The increase in immune response for Category “B” was detected by day 7 and continue to increase with highest reported value on day 21 following by almost 2-fold decrease by 6 month.

For Category “C” (re-vaccination after 30 or more years post primary vaccination) neutralizing response, from day 0 serum samples were rated as non-neutralizing with EC_50_ dilution values below a 1:40. However, similar to category “B” the immune response start to increase by day 7 (mean 112, median 97, range 40–105) and reached its peak at day 21 (mean 290, median 191, range 113–596). Observed increase in neutralizing antibodies by day 7 could be associated with the presence of long-lived memory B cells. It was reported that long-lived memory B cells can produce rapid increase in antibody secretion upon re-vaccination after 30 or more years post initial vaccination [14]. As we saw with categories “A” and “B” the amount of neutralizing antibodies was slightly lower at 6 months compared to day 21 post vaccination.

It appears that with repeated vaccinations (category “D”) the EC_50_ dilution values are lower than with primary vaccination or first time three-year boost, and do not significantly change depending on the day serum was collected. Earlier reports showed that presence of circulating neutralizing antibodies in the serum of multiple time vaccinee, in addition to strong a cellular immune response triggered by re-vaccination, leads to a fast and complete destruction of the virus at the initial stages of immune response [15, 16].

In addition to experiments with human serum, we conducted a small-scale set of tests using prairie dog serum samples from monkeypox virus challenge study [11] and compared neutralization results with previously performed ELISA data. Although two assays measure different antibody functions (neutralization vs. binding), we found a good correlation between both assays. While the results of ELISA indicate an increase in antibody presence, HC-OVNA follows the same response pattern showing neutralizing activity of tested serum, making two assays complimentary to each other.

## Conclusion

In this paper we described the development of new high content analysis infectivity and neutralization assays that are compatible with multiple species of Orthopoxviruses, and were tested with four species (vaccinia Wyeth, variola Solaimen, monkeypox MPXV-USA-2003-044, and cowpox Brighton). Both assays are based on viral immunodetection with commercially available anti-vaccinia polyclonal antibodies. Due to a high degree of genetic similarity between Orthopoxviruses, our newly-developed assays could be applied to different members of Orthopoxvirus genus utilizing commercially available polyclonal antibodies. These assays are particularly valuable for analysis of viruses which cannot be genetically modified, like variola virus, and may replace the traditional plaque and plaque reduction neutralization test (PRNT) assays. The high throughput methods possess several advantages over the traditional PRNTs: require very small amounts of test material, less labor intensive, require shorter incubation times than traditional methods. Collected images are stored on a secure server and could be re-analyzed for additional parameters associated with infection, like morphological changes in cells, variations in nucleus size, texture analysis and others. In addition, both methods could be combined with cytotoxicity and apoptosis assays to measure cellular response to a new drug and viral infection *in-vitro*, instead of, or before expensive animal model studies.
